# An Innovative Health Literacy Approach Designed to Improve Patient Understanding of Medication Labeling

**DOI:** 10.1007/s43441-021-00325-5

**Published:** 2021-08-02

**Authors:** Kara L. Jacobson, Juliette Faughnan, Laurie Myers, Amy Dubost, Lisa Courtade, Scott von Lutcken, Christine McCrary Sisk, Suzanne Gregory, Anita Cunningham, Cathryn Gunther, Michael S. Wolf

**Affiliations:** 1 Rollins School of Public Health of Emory University, 1518 Clifton Road NE, Atlanta, GA 30322 USA; 2 Sommer Consulting, Langhorne, PA USA; 3 Merck & Co., Inc., Kenilworth, NJ USA; 4 Feinberg School of Medicine, Northwestern University, Chicago, IL USA; 5 Present Address: Organon & Co., Jersey City, NJ USA

**Keywords:** Health literacy, Labeling comprehension, MedGuide, Patient labeling, Patient package insert, Patient understanding

## Abstract

**Objective:**

Limited health literacy negatively impacts understanding of medication-related information. We describe an innovative methodology designed to optimize user understanding of patient medication labeling through the systematic application of evidence-based health literacy principles, using the Patient Package Insert (PPI) for bezlotoxumab (ZINPLAVA™, Merck & Co., Inc., Kenilworth, NJ, USA) as an example.

**Methods:**

We used a mixed-model, iterative approach consisting of three phases: (1) content development; (2) focus group testing; and (3) comprehension testing. Content development was based on evidence-based health literacy principles and conducted through a collaborative partnership between industry and academia professionals. The PPI was then tested in four focus groups, two in Atlanta and two in Chicago, with an emphasis on collecting feedback from respondents with limited health literacy, evaluated using the Newest Vital Sign (NVS) health literacy assessment tool. Subsequent comprehension testing included patients with *C. diff*, caregivers, and general population members, with a pre-defined target sample of 25% with limited health literacy identified through two health literacy assessment tools: the Single Item Literacy Screener and the NVS.

**Results:**

Content development of the bezlotoxumab PPI occurred in May 2015. In June 2015, focus group respondents (*n* = 34) provided generally favorable feedback, with insights revolving around organization and usability; language and comprehension; and volume of information. Comprehension testing of the revised PPI resulted in average comprehension scores of 96% for the overall population (*n* = 59), 90% for individuals presenting with limited health literacy (*n* = 14), and 97% for those with adequate health literacy (*n* = 45). This PPI development approach was similarly effective for subsequent products across diverse therapeutic areas, with comprehension scores ≥ 86% for all participants (*n* = 1197).

**Conclusion:**

This methodology represents a significant advancement for the development of understandable patient medication labeling, especially for people with limited health literacy.

**Supplementary Information:**

The online version contains supplementary material available at 10.1007/s43441-021-00325-5.

## Introduction

The United States Department of Health and Human Services defines health literacy as “the ability to obtain, process, and understand health information to make appropriate health decisions” [1]. It is estimated that approximately one-third of adults in the United States have limited health literacy [2]. The sociodemographic factors associated with limited health literacy include advanced age, low income, racial and ethnic minority status, limited education, limited English proficiency, and cognitive/functional impairments [3]. People from many of these sociodemographic groups are at an increased risk for chronic health conditions, requiring continued use of multiple prescription medications. At the same time, people with limited health literacy are disproportionately at risk for poorer medication adherence [4], which can lead to adverse drug reactions, higher hospitalization rates, increased medical costs, poor health outcomes, and increased mortality [5]. This is likely related, at least in part, to patient misunderstanding of information about their prescribed medications [6–8]. These deleterious effects signal an urgent need for improved health communication, empowering patients to engage in shared decision-making and effectively manage their health.

To support safe and effective drug use, many countries have regulatory requirements governing medication labeling to ensure that the information is clear and accessible for patients, including those with limited health literacy [9–12]. In the United States, the Food and Drug Administration (FDA) reviews and approves plain-language patient labeling for certain products to be distributed directly to patients by their health care providers. One type of labeling is the Patient Package Insert (PPI), which is required in the United States for select prescription medications (e.g., oral contraceptives and estrogens) and may also be provided voluntarily by manufacturers for other drugs [13]. The PPI includes details about a drug’s indication, dosage, route of administration, instructions for use, warnings, and contraindications. However, the available research suggests that consumers experience substantial difficulty understanding PPIs [14–16] and this is particularly problematic for people with limited health literacy [16–18]. Of great concern, many people with limited health literacy do not understand the warnings and potential side effects associated with their prescribed medications [19].

Given the volume and complexity of prescription drug information, it is critical to ensure that all patients understand the information about their medications, which, in turn, may be expected to reduce medication errors, increase adherence, and promote patient health and safety. Over recent years, the FDA has sought guidance and participation from academia, patient advocacy groups, and the pharmaceutical industry to innovate and develop new strategies to improve patient understanding of drug information. The field of health literacy research has been a valuable resource for identifying ways to help people access and understand written medication materials [20, 21]. The objective of this report is to describe an innovative methodology designed to optimize patient understanding of the PPI through the systematic application of evidence-based health literacy principles [22].

## Materials and Methods

### Study Design

In the present report, the PPI for bezlotoxumab (ZINPLAVA™, Merck & Co., Inc., Kenilworth, NJ, USA), a medication to treat *Clostridium difficile* (*C. diff*) infection, which can damage one’s colon and cause stomach pain and severe diarrhea, was selected as an example to fully illustrate the health literacy development process. To inform the reproducibility of results using this methodology, comprehension testing scores of PPIs for multiple medications across diverse therapeutic areas are summarized.

**Figure 1:**
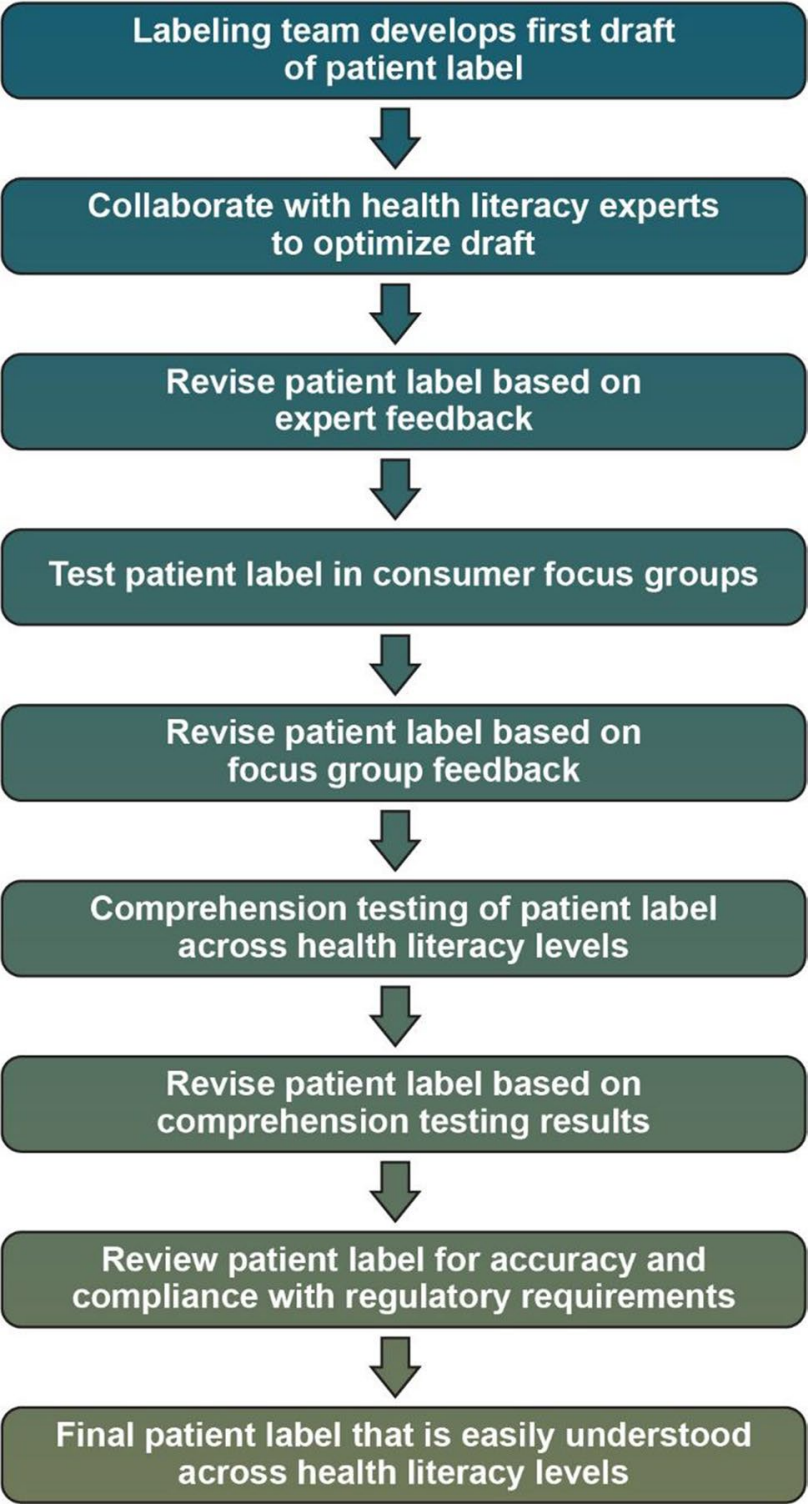
Health Literate Patient Medication Labeling Process Map.

**Figure 2:**
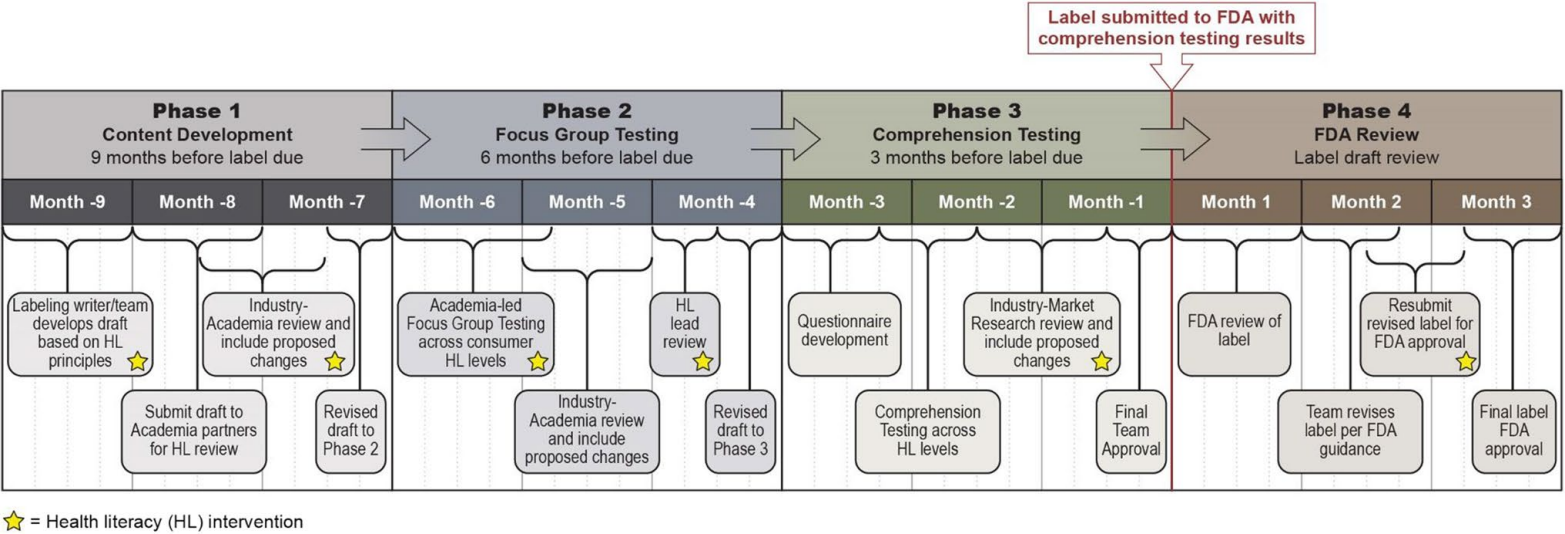
Health Literate Labeling Process Timeline.

A three-step, mixed-model, collaborative, iterative, health literacy PPI development methodology was used: (1) content development; (2) focus group testing; and (3) comprehension testing (Fig. 1). An example process timeline is illustrated in Fig. 2. The objectives of this approach are to determine the optimal way to present information to maximize comprehension; to understand consumers’ interpretation of key areas of importance in the PPI, including content, format, layout, order, emphasis, and language; and to identify areas of confusion or concern and implement the necessary revisions to improve PPI comprehension.

### Phase 1: Content Development

The first draft of the bezlotoxumab PPI was developed by a professional medical writer who had received prior training in health literacy principles and best practices (Health Literacy Media, St. Louis, MO, USA) [22]. At the earliest stages of PPI development, health literacy principles concerning planning, content and organization, layout and typography, and graphics were implemented (Table 1). These principles were developed to better align health information with the skills and abilities of those using that information.

**Table 1:** Health Literacy Principles Checklist

*Planning*	
Objective	∙ Define the communication objectives
Target audience	∙ Know audience need, interests, and behaviors
	∙ Identify ways to engage the target audience
	∙ Involve the target audience in development and testing
*Content*	
Purpose	∙ Focus and limit the objectives
	∙ State objectives in the title, cover illustration, and introduction
Evidence	∙ Ensure content is accurate and evidence based
	∙ State what is known and when relevant, what is not known
Scope	∙ Limit to essential information; include the “need to know,” but not the “nice to know”
	∙ Include only information that is relevant and meaningful to the intended audience
	∙ Focus on behaviors, skills, and instructions
	∙ Go beyond the facts to include action-oriented material
	∙ Stress, repeat, and summarize the main points
Language and culture	∙ Ensure high-quality translation and interpretation of content
Demographics	∙ Ensure content reflects age, education, income, gender, occupation, and residence of intended audience
Clarity	∙ State the information as clearly and simply as possible
Tone and appeal	∙ Include positive, truthful, and helpful content
	∙ Edit content for bias and prejudice
References	∙ Note key sources
	∙ Provide sources for more information
Date/authorship	∙ Include author(s) and date of publication or revision
*Literacy demands*	
Reading level	∙ Ensure as many people as possible can read and understand the materials
	∙ Consider using a readability calculator, but be sure you understand its limitations
Choice of words	∙ Use common, everyday, specific words that are two syllables or less
	∙ Avoid using jargon, abstract words, technical terms, statistics, abbreviations, and acronyms
	∙ Include the pronunciation of words that may not be familiar
	∙ Explain words, expressions, and phrases through simple definitions; consider using word/picture association or examples
	∙ Use positive statements. Limit sentences that begin with “don’t” or “never”
Sentences/paragraphs	∙ Write in a conversational style, using active voice
	∙ Use short, simple, and direct sentences (8–15 words)
	∙ Use short paragraphs and avoid large blocks of text
Use of numbers	∙ Do the math for the reader, do not require addition, subtraction, multiplication, or division
	∙ Consider using visual presentations of numbers
	∙ Use whole numbers when possible (1 in 1,000 instead of 0.001)
	∙ Express risk information in frequencies (1 out of 10 instead of 10%)
*Organization*	
Content	∙ Present context before new information
Sequence	∙ Present information that is logical and easy to follow
	∙ Position important information at the beginning and end for emphasis
Groupings	∙ Divide information into small, logical sections
Headings	∙ Use short, simple, and explanatory headings to organize
*Layout and typography*	
Font	∙ Use a clear typeface like Times New Roman or Arial
	∙ Use upper and lowercase letters; avoid using all uppercase
	∙ Limit the variations of typefaces used
	∙ Use a type size that is easy to read and as large as possible (at least 12-point; 14-point; or 16-point text is preferred)
Headlines and titles	∙ Use headlines and titles to orient and engage the reader
White space	∙ Provide breaks for the eye with white space
	∙ Balance the use of white space with content and graphics
	∙ Separate paragraphs and topics by one or two lines
Justification	∙ Avoid full justification of text; format edges flush left with right ragged instead
Highlights and color	∙ Use bulleted lists, underlining, and bold for emphasis
	∙ Introduce color to highlight, add clarity, differentiate, or focus the eye
	∙ Use shading, boxes, and arrows to direct attention to key content
*Graphics*	
Engagement	∙ Use graphics to grab attention
	∙ Spotlight the objectives with the cover graphic
	∙ Reinforce the objectives with graphics
	∙ Choose graphics that are friendly, attractive, and recognizable
Relevancy	∙ Use action-oriented graphics
	∙ Show key desired behaviors, rather than behaviors to avoid
	∙ Choose graphics that reflect the age, gender, ethnic, and cultural background of the audience
	∙ Design graphics that support and reinforce important points
Clarity	∙ Use simple design, free from clutter and distractions
	∙ Avoid diagrams, graphs, charts, and data tables that require multiple steps for use

Doak et al. [23]

Once completed, the PPI draft was circulated by the medical writer through an open-access cross-functional review process, which included representatives from the following departments of the pharmaceutical manufacturer (Merck & Co., Inc., Kenilworth, NJ, USA): Product Labeling, Clinical Development, Regulatory Affairs, Global Clinical Safety and Risk Management, Clinical Research, Marketing, Market Research, Legal, and Health Literacy. The PPI was revised by the medical writer in accordance with the comments from each stakeholder.

Following content approval consensus, the PPI draft was submitted by the medical writer for critical review by health literacy experts from academia (Emory University, Atlanta, Georgia [K.L.J.] and Northwestern University, Chicago, Illinois [M.S.W.]) who applied evidence-based health literacy best practices based on vast experience working with people across a range of health literacy levels [22]. Further revision of the PPI was conducted through an open-access, iterative process, which included experts from both the pharmaceutical manufacturer and the academic institutions. Following this process and approval from all stakeholders, the resulting version of the PPI draft was moved forward to Phase 2 of the process: focus group testing.

### Phase 2: Focus Group Testing

Independent focus group testing was conducted by the academic partners and health literacy experts from Emory (K.L.J.) and Northwestern (M.S.W.) Universities. Representatives of the pharmaceutical company neither observed nor participated in the focus groups. Four focus groups were conducted, two in Atlanta and two in Chicago. In Atlanta, a convenience sample of patients was recruited from the outpatient primary care clinics of Atlanta’s inner-city indigent hospital, where patients have been shown to have limited health literacy skills [24]. Patients were approached and the study methods were explained (i.e., they were being invited to return to the hospital on a different day and time to participate in a focus group discussion). Patients received a phone call to remind them of their focus group meeting day, time, and location. In Chicago, participants were recruited through Craigslist. The focus group participants were from the general population (i.e., people who did not have *C. diff*) to ensure that they had no specific prior knowledge of the disease. The population was heavily skewed towards having limited health literacy, which was evaluated using the Newest Vital Sign (NVS) assessment tool, a previously validated measure of health literacy with high sensitivity for detecting people with limited health literacy [25]. The focus group methodology was reviewed and approved by the Institutional Review Boards at both participating academic institutions. All research participants provided written informed consent, and at the end of the discussion, they were provided $40.00 in grocery store gift cards for their time.

A facilitator’s topic guide (Online Resource 1. Supplementary Materials) was developed to consistently assess participants’ overall comprehension of the PPI and collect feedback about the layout and design across sites. The focus group participants discussed the strengths and weaknesses of the bezlotoxumab PPI and provided suggestions for improvement. Specifically, participants were asked about why this medicine would be prescribed for a patient; how this medicine is different from antibiotics; what are the side effects of the medicine; and if they had any suggestions for improvements in content, logical structure, and level of detail.

The focus group discussion results were reviewed, and the final recommendations formed the basis for further refinement of the PPI using the same academia–industry iterative process described for Phase 1. The resulting version of the PPI was forwarded to Phase 3 of the process: comprehension testing.

### Phase 3: Comprehension Testing

The interview flow for comprehension testing is illustrated in Table 2. Comprehension testing of the PPI was conducted by an independent marketing research firm (J.F., Sommer Consulting, Langhorne, Pennsylvania, USA). A sample size target of approximately 60 participants was planned, and included patients who had been treated or were currently being treated for *C. diff* infection (*n* = 24), caregivers who were primarily responsible or actively involved in the treatment decisions of patients diagnosed with *C. diff* (*n* = 12), and general population members who did not have *C. diff*, but had similar demographics (e.g., age, ethnicity, and gender) to patient populations with the highest incidence of *C. diff* infection (*n* = 24). General population participants were included to ensure that even people without prior knowledge of the disease state could understand the information presented in the bezlotoxumab PPI. As *C. diff* is more prevalent in older people, we ensured that most patients and general population participants were aged 65 and older.

**Table 2:** Comprehension Testing Interview Flow

General usage: Consumers were asked a general set of questions regarding patient labeling use, including when and if they read patient labeling information, their primary language, etc
Review of patient labeling: Consumers read the patient labeling in its entirety at their own pace, which was timed to ensure it fell within the acceptable parameters of five minutes. On average, it took respondents 2.5 min to read the proposed labeling
Comprehension test (open-book questions): Patient labeling remained with the consumer. The untimed 14-question test consisted of a mix of multiple choice and true/false questions. Consumers could refer to the patient labeling at any time to find the answers
Patient labeling deep dive (open-book review): Patient labeling was reviewed with consumers in detail to gain a greater depth of understanding of their comprehension. Specific attention was paid to questions answered incorrectly during the comprehension test. Consumers were asked to identify what they believed caused those questions to be answered incorrectly (e.g., they forgot the information, missed/skipped over the information, were confused by the information, misunderstood the information, etc.)
Utilization and recall (closed-book review): Patient labeling was removed and consumers were then asked open-ended questions to assess their understanding of the medication, what it is for, how it should be taken, what important safety information they should tell their doctors, and possible side effects
Newest Vital Sign (NVS) assessment: The NVS Health Literacy Exam was administered to consumers following the interview to confirm their health literacy levels. This measurement was used to segment Limited and Adequate Health Literacy respondents

Research participants were recruited from the Schlesinger national database (Schlesinger Group, Iselin, Woodbridge Township, NJ) either by telephone or online channels and supplemented with participants from literacy centers and senior centers to derive a diverse study sample, representative of broad age groups and health literacy levels. Based on feedback from external health literacy experts and assessment of the feasibility to recruit, a goal of at least 25% of the population with limited health literacy in the total sample was pre-specified. Health literacy levels were assessed during an initial screening process using the validated Single Item Literacy Screener (SILS): “How confident are you filling out medical forms by yourself?” [26]. Prior research has shown that this instrument has a 0.74 to 0.84 area under the receiver operating characteristic curve (p < 0.05) with performance-based, assessed health literacy levels [26]. This single question allowed for the rapid assessment of health literacy levels of potential participants to support adequate representation of limited health literacy respondents in the study sample. The final classification of respondents by health literacy level was confirmed at the end of comprehension testing using the Newest Vital Sign (NVS) assessment tool [25]. Respondents were classified into one of two categories: limited health literacy (NVS score, 0–3) or adequate health literacy (NVS score, 4–6).

The comprehension test was developed by the marketing research firm (Sommer Consulting) and was composed of multiple choice and true-or-false questions based specifically on the bezlotoxumab PPI. The comprehension test assessed the following topics: (1) what bezlotoxumab is used for and how it is used in addition to an antibiotic to treat *C. diff* infection; (2) the common side effects associated with taking bezlotoxumab; (3) what patients should tell their doctor before starting bezlotoxumab; (4) how bezlotoxumab is administered; and (5) how long the administration process takes. Comprehension testing entailed a mix of in-depth, qualitative 45- to 60-min interviews conducted either in-person or by phone/Web (materials were displayed via the Internet, with hard copies sent to respondents without computer access). Both open-book (i.e., allowing participants to view the material when responding to questions) and closed-book (i.e., asking participants to respond to questions after the material has been removed) approaches to comprehension testing were used. The participant’s comprehension score was calculated as the percentage of correct answers on the test, with a goal score of at least 80% comprehension across all consumer segments, prospectively established based on a U.S. Elementary School-level grading system, in which 80% accuracy is equivalent to a “B” grade [27]. In addition, all incorrect answers were probed to determine the reason (e.g., the information could not be found, the participant misunderstood the information, there was contradictory information, etc.) in order to guide changes that truly increase comprehension of the PPI. Based on the comprehension testing results, the draft PPI was further revised before finalization, as necessary.

A summary of the comprehension testing results from participants across health literacy levels was subsequently submitted to the FDA along with the final PPI for informational and approval purposes. Although not a regulatory requirement, the manufacturer believed this would be helpful insight for the FDA to consider during approval of the patient labeling.

## Results

### Phase 1: Content Development

Following initial content development based on established health literacy principles, the bezlotoxumab PPI draft was provided by the drug manufacturer to health literacy experts at Emory University (K.L.J.) and Northwestern University (M.S.W.) for content review in May 2015. The guiding criteria used for the further refinement of the draft PPI were derived from the Institute of Medicine discussion on health literacy principles, which includes such factors as content, organization, layout, and graphics [22].

### Phase 2: Focus Group Testing

Focus group testing of the bezlotoxumab PPI occurred in June 2015 with 34 participants, 16 from the Atlanta site and 18 from the Chicago site. The focus group feedback was generally favorable; respondents indicated that the bezlotoxumab PPI was “very clear,” “easy to read,” “thorough,” and “informative,” with specific insights revolving around three overarching categories: (1) organization and usability; (2) language and comprehension; and (3) volume of information (Table 3). One noteworthy insight from the focus group feedback was the need for greater clarification that bezlotoxumab is not an antibiotic medication and it is therefore important to continue to take prescribed antibiotic medications for *C. diff* as directed by the physician (Table 3).

**Table 3:** Focus Group Feedback*

1. Organization and usability
a. Most believed the information followed a logical order
b. The picture of the IV was considered “helpful.” Some said they did not need the picture themselves, but other respondents were not certain of the abbreviation “IV” and they found the illustration very helpful
2. Language and Comprehension
a. There was a need for greater explanation that bezlotoxumab is not an antibiotic and it is therefore important to continue taking prescribed antibiotic medications for *C. diff*
b. The language was “easy to understand” and not too technical or medical
c. Section titles, bullets, and bolding made the key ideas stand out and the information “easy to find.”
3. Amount of Information
a. Most indicated it was “thorough” yet “not overwhelming.”
b. Some noted they would likely read through it since it is “short and sweet.”
Reasons for favorable responses:
Headings: Informative headings drew attention to important information and helped readers navigate through the document and locate key points
Bullets: The bullets made it easy to sort through the document, and information that followed was concise. The bullets were eye-catching and signified importance
Spacing: The white space pleased the eye. The text was less overwhelming
Bolding: The bolding emphasized the important sections; it simplified skimming and helped respondents to reference back to the document
Font: The use of a clean, 12-point font simplified reading flow
Graphic: The picture of the IV enhanced understanding by supporting the text with relevant imagery

*Approved drug label may not reflect all focus group feedback due to revisions made during the FDA review process

### Phase 3: Comprehension Testing

Comprehension testing was conducted from August to September 2015 with 59 participants: 24 patients with *C. diff*, 11 caregivers of patients with *C. diff*, and 24 members of the general population who did not have *C. diff* but closely matched the demographic profile of a newly diagnosed patient (Table 4). Overall, 24% of participants were classified as having limited health literacy according to the NVS assessment (Table 5).

**Table 4:** Bezlotoxumab Comprehension Testing Interview Demographics (Actual/Target) by Participant Segment and Age, Ethnicity, and Gender Groups

	General Population Actual/Target	Patients with *C. diff* Actual/Target	Caregivers of Patients with *C. diff* Actual/Target
Age			
18–64	6/4–5	6/4–5	5/2–3
65–74	16/14–16	12/14–16	6/6–8
75+	2/4–5	6/4–5	0/2–3
Ethnicity			
Caucasian	19/17–18	22/17–18	8/8–9
Hispanic/Latino	3/3–4	1/3–4	1/1–2
African American	2/1–2	1/1–2	2/1–2
Other	0/1–2	0/1–2	0/1–2
Gender			
Female	14/15	15/15	7/7
Male	10/9	9/9	4/5
Total	24/24	24/24	11/12

**Table 5:** Health Literacy Level of Comprehension Testing Interview Sample

Patients with *C. diff* (*n* = 24)		Caregivers of Patients with *C. diff* (*n* = 11)		General Population (*n* = 24)		Total Limited	Total Adequate
Limited	Adequate	Limited	Adequate	Limited	Adequate	14	45
7	17	1	10	6	18		
24		11		24		59	

**Figure 3:**
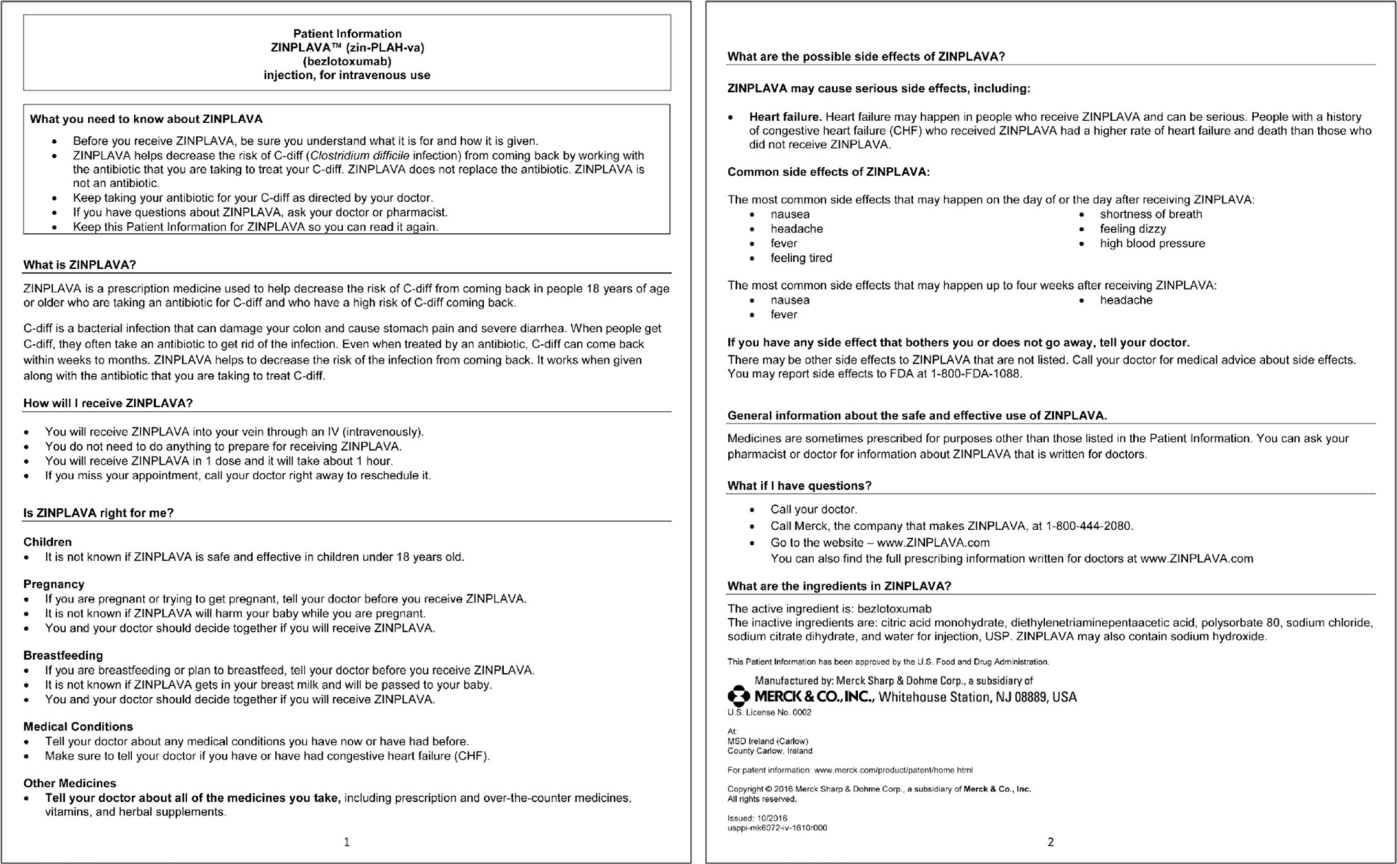
FDA Approved Bezlotoxumab Label.

The average participant comprehension of the bezlotoxumab PPI across all demographic categories, including audience, education level, and age, was 96%, far exceeding the pre-specified > 80% comprehension score goal. As shown in Table 6, participants with both limited and adequate health literacy had high comprehension scores (90% and 97%, respectively). In the utilization and recall part of comprehension testing (i.e., the closed-book review), most respondents were able to recall key information from the PPI and answer specific questions correctly after the PPI was removed. Ultimately, this approach resulted in a final PPI for bezlotoxumab with high participant comprehension, which was approved by the FDA and issued in October 2016 (Fig. 3).

**Table 6:** Comprehension Testing Results for Bezlotoxumab

*Average Comprehension Score by Audience Group*											
Patients with *C. diff* (*n* = 24)		Caregivers of Patients with *C. diff* (*n* = 11)			General Population (*n* = 24)		Adequate Health Literacy (*n* = 45)			Limited Health Literacy (*n* = 14)	
97%		94%			95%		97%			90%	

*Average Comprehension Score by Education Level*											
High School Graduates (*n* = 7)			Some College (*n* = 22)			College Graduates (*n* = 18)			Post-graduate (*n* = 12)		
97%			95%			97%			93%		

*Average Comprehension Score by Age*											
Ages 18–34 (*n* = 3)	Ages 35–44 (*n* = 3)			Ages 45–54 (*n* = 4)		Ages 55–64 (*n* = 7)		Ages 65–74 (*n* = 34)			Age 75 + (*n* = 8)
98%	100%			91%		99%		95%			96%

### Summary Results of Comprehension Testing Across Multiple Medications

Since 2015, this process of PPI development has been applied to 17 different drug products across diverse therapeutic areas (*n* = 1197). The results consistently demonstrate that this approach optimizes comprehension of PPIs across participant health literacy levels, with average comprehension testing scores ≥ 86% across all products and population segments studied (Table 7; Online Resource 2; Supplementary Materials).

**Table 7:** Aggregate Results Across Indications

Indication	Sample	Average Comprehension Score		
		Overall	Health Literacy Level	
			Low	Adequate
*C. diff*	60^a^	98%	97% (*n* = 12)	99% (*n* = 47)
HIV-1	60	97%	96% (*n* = 22)	98% (*n* = 38)
Acute bacterial skin and skin structure infections (ABSSSI)	60	96%	95% (*n* = 16)	96% (*n* = 44)
CMV infection	50	98%	96% (*n* = 14)	98% (*n* = 36)
Type 2 Diabetes	60	94%	92% (*n* = 12)	94% (*n* = 48)
HIV	45	90%	90% (*n* = 18)	90% (*n* = 27)
*C. diff*	59	96%	90% (*n* = 14)	97% (*n* = 45)
Hepatitis C	60	96%	92% (*n* = 17)	97% (*n* = 43)
Osteoporosis	101	96%	92% (*n* = 30)	97% (*n* = 71)
Melanoma	60	92%	86% (*n* = 22)	95% (*n* = 38)

^a^One participant was unable to take NVS test due to poor vision; for this participant, health literacy level was reported as “unassigned.”

## Discussion

In the present report, we describe an innovative health literacy approach to the development and testing of the PPI using a collaborative, mixed-model, three-step process. This approach ultimately resulted in PPIs that are associated with high average comprehension scores across all health literacy levels, with the pre-defined goal of achieving comprehension scores of at least 80% exceeded across all population segments tested. By comparison, a recent study by Patel and colleagues examined comprehension of standard or health literacy-friendly drug summaries among cohorts of patients with adequate and low literacy. The authors reported that comprehension improved from 42% to only 52% among low literacy patients [28], much lower than the 90% level of comprehension observed using our approach to developing health literate PPIs. Notably, 71% of respondents in our study were aged 65 years and older, a sample representative of patients who would use medications to treat *C. diff* infection, which is prevalent among older populations.

### Phase 1: Content Development

During content development of the bezlotoxumab PPI, evidence-based health literacy principles were applied during initial drafting and the entire revision process. The comprehensive cross-functional review process allows for the inclusion of broad perspectives across multiple stakeholders from the pharmaceutical manufacturer. These reviewers include representatives from Product Labeling to ensure that the information is accurate and informative and from Market Research to plan analyses to discover consumer informational needs and preferences. With the recognition that all communication to patients about a drug product must be consistent with the approved product labeling, the Legal review is integral to confirm that the PPI is not promotional or misleading, makes no implied claims without supporting evidence, and is based on data derived from human experience. From this perspective, incorporating health literacy principles from the earliest stages of product labeling is an important best practice, as it is challenging to revoke information following regulatory approval and doing so may have significant product liability implications. Representatives from Marketing participate to understand the labeling information that will be available after product approval.

The content development process is unique insofar as it is conducted through a collaborative partnership between industry and academia. Following review and approval by stakeholders within the pharmaceutical manufacturer, the draft PPI undergoes critical review and revision by patient-facing health literacy experts from academic institutions through an iterative process, with a goal of making the PPI increasingly health literate with each iteration. Importantly, there is a commitment to implement the suggestions from academia partners, unless the edits make the information medically inaccurate or incomplete. Overall, the content development phase ensures PPI optimization very early in the process, even before qualitative market research with patients/consumers.

### Phase 2: Focus Group Testing

During the focus group testing phase of development, direct patient/consumer input is rigorously solicited and incorporated into the draft PPI. For bezlotoxumab, focus group participants included patients from an inner-city, indigent hospital outpatient clinic and consumers from a large urban city, settings expected to provide a representative sample of people with limited health literacy. The focus groups included 16 participants from the Emory, Atlanta site and 18 participants from the Northwestern, Chicago site, for a total of 34 focus group testing participants. The focus group feedback suggested that the PPI was generally understandable and informative; however, one important source of confusion emerged: there was a need to clarify that bezlotoxumab is not an antibiotic medication and patients must therefore continue to take prescribed antibiotics for *C. diff* as directed. This insight has serious safety implications and would not have been considered without focus group testing, highlighting the importance of including the patient/consumer perspective in the labeling development process.

### Phase 3: Comprehension Testing

Our former approach to PPI development consisted of comprehension testing across a range of education levels; however, health literacy assessments revealed underrepresentation of patients with limited health literacy. Thus, the current process of comprehension testing now incorporates several key enhancements. Deliberate efforts to include research participants who closely match the target patient population are made whenever appropriate based on the disease state; recruitment of participants from the patient/consumer sector focus specifically on identifying robust (approximately 60 participants) and representative (diagnosed patients, caregivers, and demographically matched members from the general population) samples, with a pre-specified goal to include ~ 25% of participants with limited health literacy. The segment with limited health literacy is identified through validated health literacy screening methodology, using two separate screening tools: the SILS and the NVS. Compared with traditional readability formulae based solely on word length or syllable count, these health literacy assessment tools consider other measures, including structure, content, coherence, and design factors.

For a variety of reasons, including fear or embarrassment of literacy abilities [29], lack of transportation to the research site, and chronic health conditions, it is challenging to recruit research participants with limited health literacy. Due to these challenges, additional recruitment time is factored into the development timeline to allow for recruitment efforts through more inclusive means, such as onsite, in-person grassroots recruiting at literacy centers and senior centers, which take longer than working with the convenience sample available in standard recruitment databases.

For comprehension testing of the PPI, the in-person interview format is effective for respondents with limited health literacy who may be hesitant to participate in research due to an inability to complete a questionnaire or who do not have access to technology. It also allows members of the PPI development team, including representatives from Product Labeling who would not have traditionally participated in comprehension testing, to observe interviews, identify visual cues, and assess respondents’ level of understanding of the materials. The phone/Web interview format allows for a broad, diverse geographic dispersion and reach.

The use of both open-book and closed-book approaches to comprehension testing is consistent with health literacy and cognitive factors research recommendations, indicating that the task under evaluation should most accurately mimic what would be expected of a person during actual use. Allowing consumers to review and re-review content in the open-book approach is representative of real-world experience, since the PPI and other forms of drug information are external aids to which patients may refer as needed. The purpose of the closed-book approach is to determine recall and ensure understanding of key information; particularly, important side effects and when to contact a health care provider.

### Summary Results of Comprehension Testing Across Multiple Medications

The current process has been applied to subsequent development and testing of multiple PPIs. Our approach has been similarly effective for drugs across many diverse therapeutic areas, as evidenced by high comprehension scores among participants with both adequate and limited health literacy. Additionally, this methodology has been successfully used to develop other types of patient labeling, specifically Medication Guides and Instructions for Use. The reproducibility of results suggests that this approach is an optimal model for the development of patient labeling.

### Research Strengths and Limitations

To our knowledge, our process is the first mixed methods, health literacy approach to PPI development based on a collaborative partnership between industry professionals and health literacy experts from academia. Key strengths of this approach are the inclusion of multiple perspectives, particularly those of the patient/consumer, and incorporation of evidence-based health literacy principles and best practices throughout the entire process. Comprehension testing of the PPI is supported by a sample that is robust and representative of the patient population across health literacy levels. Indeed, the inclusion of 25% of participants with limited health literacy identified by two validated assessment instruments is a key strength. Finally, the reproducibility of results across multiple products in different therapeutic areas permits further confidence in the validity of this approach.

The present research also has some limitations. The focus group results are based on a small sample of participants from large, urban US cities and may not be generalizable to different sociodemographic populations or countries. However, the comprehension testing phase recruits from a nationwide sample of urban, suburban, and rural areas across the country to ensure a mix of respondents. Further, only English-speaking (or English as their second or later language) participants were enrolled, as the PPI was written in English. Future research should consider a broader audience, including those from other regions and who do not speak English, especially considering that low English proficiency correlates with poor comprehension of health information. In addition, because there are many different health literacy assessment tools, classification of participants by health literacy level may vary between instruments. To help address this concern, two validated health literacy assessment tools, the SILS and the NVS, were used simultaneously to classify participants by health literacy level.

## Conclusions

The present health literacy approach represents a significant advancement in the development of prescription drug PPI. The success of this approach is demonstrated by high comprehension across all consumer segments, including people with limited health literacy.

## Practical Implications

In practice, this approach to PPI development empowers patients to better manage their own health by facilitating an improved understanding of their prescribed medications. This approach is expected to culminate in greater medication adherence, fewer adverse drug reactions, and improved health outcomes.

## Future Directions

Additional research is needed to examine how the implementation of these best practices will improve patient knowledge and compliant use of prescription medications. This methodology may be expanded to other types of consumer health research and adopted by health care organizations and regulatory agencies to support the development of patient drug information that is widely understood across diverse populations and health literacy levels. The adoption of health literacy as a key advocacy initiative is needed to improve the understanding of medication information for all patients.

## Supplementary Information

Below is the link to the electronic supplementary material.

Supplementary file1 (PDF 253 kb)
