# Supplementary material for: An Innovative Health Literacy Approach Designed to Improve Patient Understanding of Medication Labeling
Source: Ther Innov Regul Sci. 2021 Aug 2;55(6):1180–92. doi: 10.1007/s43441-021-00325-5 (PMC8492593; doi:10.1007/s43441-021-00325-5)
Supplement: Supplementary file 1 — Supplementary file1 (PDF 253 kb) [file 43441_2021_325_MOESM1_ESM.pdf]

Supplementary Material for: An Innovative Health Literacy Approach Designed to Improve Patient Understanding of Medication Labeling

Kara Jacobson, Juliette Faughnan, Laurie Myers, Amy Dubost, Lisa Courtade, Scott von Lutcken, Christine McCrary Sisk, Suzanne Gregory, Anita Cunningham, Cathryn Gunther, Michael S. Wolf

Therapeutic Innovation & Regulatory Science

Corresponding author:

Kara L. Jacobson, MPH

Rollins School of Public Health of Emory University

1518 Clifton Road, NE Room 668

Atlanta, GA 30322

E-mail: kljacob@emory.edu

Online Resource 1. Bezlotoxumab PPI Focus Group Discussion Topic Guide

1. **In general, how helpful is print material that you may get for a prescribed drug, whether it is from your doctor or the pharmacy? Do you get material anywhere else?**
   A. How do you use these materials?
   B. Where do you keep them?
   C. How often do you refer to them?
2. **The PPI that we are going to discuss today is for a drug that is so new it does not yet have a name, so you'll just see "trade name" in place of the actual name. But the information is not made up; it's for this new drug. You all have a copy in front of you, so my questions from this point forward will be about this medicine specifically.**
   A. Take a moment to review – what are your first impressions?
   B. We really want to know how to make this better. Starting with the general appearance, how clear is this – in other words, does it appear to be easy to read?
   C. If you were prescribed this drug, what is the first thing you would want to know? What do you think about the order of information here? Would you change it?
   D. Are there any terms or phrases that are confusing?
   E. I know this may be hard to determine, but can you think of any image or table or chart that would be helpful here that we could add?
   F. How about the font, is this attractive? Is it easy to read?
   G. In general, are there any recommendations you have for making this document better?

H. Would you keep this? Would you refer to this again? Do you think this is better than what materials you have seen? Why or Why not?

By section:

- **‘What is ZINPLAVA’**
- **‘What should I tell my doctor before taking ZINPLAVA?’**
- **‘What are the possible side effects of ZINPLAVA?’**
- **‘How do I take ZINPLAVA?’**
- **‘What if I forget to take ZINPLAVA’**
- **‘How should I keep/store ZINPLAVA?’**
- **‘What if I have Questions?’**

Other:

- **What do we get rid of?**
- **What should go on the front page?**
- **Where do you go for information if questions arise?**

Online Resource 2. Combined patient labeling studies

**Health Literacy Levels Across Audience Group** (n=1197)*				
	**Patients** (n=505)	**Caregivers** (n=294)	**Gen Pop** (n=398)†	**Overall** (n=1197)
**Limited Health Literacy**	120	49	162	331
**Adequate Health Literacy**	385	245	236	866

*Includes testing for 16 Med Guides/PPIs.

†Two Gen Pop respondents had unassigned health literacy levels, as they were visually impaired and could not see the NVS label.

**Average Comprehension Scores Across Health Literacy Levels** (n=1197)*				
	**Patients** (n=505)	**Caregivers** (n=294)	**Gen Pop** (n=398)†	**Overall** (n=1197)
**Limited Health Literacy**	90%	92%	91%	91%
**Adequate Health Literacy**	95%	96%	95%	95%
**Overall**	94%	95%	93%	94%

*Includes testing for 16 Med Guides/PPIs.

†Two Gen Pop respondents had unassigned health literacy levels, as they were visually impaired and could not see the NVS label.

**Average Comprehension Scores Across Patient Labeling Studies** (n=1195)*		
**Health Literacy Level**	**NVS Score**	**Average Comprehension**
Limited	0 (n=39)	89%
Limited	1 (n=54)	90%
Limited	2 (n=93)	90%
Limited	3 (n=144)	92%
Adequate	4 (n=205)	92%
Adequate	5 (n=320)	95%
Adequate	6 (n=340)	97%

NVS, Newest Vital Sign.

*Includes testing for 16 Med Guides/PPIs.

**Health Literacy Levels Across Education Levels** (n=1178)*						
	**Some High School** (n=58)	**Completed High School** (n=170)	**Some College** (n=397)	**Completed College** (n=363)	**Some Post Grad** (n=39)	**Completed Post Grad** (n=151)
**Limited Health Literacy**	35	79	103	91	3	17
**Adequate Health Literacy**	23	91	294	272	36	134

*Includes testing for 16 Med Guides/PPIs.

**Average Comprehension Scores Across Education Levels** (n=1178)*						
	**Some High School** (n=58)	**Completed High School** (n=170)	**Some College** (n=397)	**Completed College** (n=363)	**Some Post Grad** (n=39)	**Completed Post Grad** (n=151)
**Limited Health Literacy**	88%	90%	90%	93%	86%	92%
**Adequate Health Literacy**	90%	95%	95%	95%	96%	96%
**Overall**	89%	93%	94%	95%	96%	95%

*Includes testing for 16 Med Guides/PPIs.

**Health Literacy Levels Across Age Levels** (n=1110)*						
	**Ages 18-34** (n=247)	**Ages 35-44** (n=177)	**Ages 45-54** (n=243)	**Ages 55-64** (n=236)	**Ages 65-74** (n=168)	**Ages 75+** (n=39)
**Limited Health Literacy**	70	40	86	64	45	15
**Adequate Health Literacy**	177	137	157	172	123	24

*Includes testing for 16 Med Guides/PPIs.

**Average Comprehension Scores Across Age Levels** (n=1110)*						
	**Ages 18-34** (n=247)	**Ages 35-44** (n=177)	**Ages 45-54** (n=243)	**Ages 55-64** (n=236)	**Ages 65-74** (n=168)	**Ages 75+** (n=39)
**Limited Health Literacy**	91%	93%	91%	91%	91%	84%
**Adequate Literacy**	95%	96%	95%	95%	96%	97%
**Overall**	94%	95%	94%	94%	95%	92%

*Includes testing for 16 Med Guides/PPIs.

Health Literacy Levels Across Ethnicities (n=1193)*						
	**Caucasian** (n=763)	**Non-Hispanic African American** (n=299)	**Hispanic** (n=88)	**Hispanic African American** (n=2)	**Asian** (n=31)	**Other** (n=10)
**Limited Health Literacy**	155	136	27	2	7	3
**Adequate Health Literacy**	608	163	61	0	24	7

*Includes testing for 16 Med Guides/PPIs.

Average Comprehension Scores Across Ethnicities (n=1193)*						
	**Caucasian** (n=763)	**Non-Hispanic African American** (n=299)	**Hispanic** (n=88)	**Hispanic African American** (n=2)	**Asian** (n=31)	**Other** (n=10)
**Limited Health Literacy**	91%	91%	89%	92%	91%	94%
**Adequate Literacy**	96%	93%	93%	-	96%	97%
**Overall**	95%	92%	92%	92%	95%	96%

*Includes testing for 16 Med Guides/PPIs.
